# 5-a-day fruit and vegetable food product labels: reduced fruit and vegetable consumption following an exaggerated compared to a modest label

**DOI:** 10.1186/s12889-018-5528-0

**Published:** 2018-05-15

**Authors:** K. M. Appleton, H. J. Pidgeon

**Affiliations:** 1 Research Centre for Behaviour Change, Department of Psychology, Bournemouth University, Bournemouth, BH12 5BB UK; 2 Research Centre for Behaviour Change, Department of Psychology, Bournemouth University, Poole House, Fern Barrow, Poole, BH12 5BB UK

**Keywords:** Fruit and vegetables, 5-a-day, Labels, Intake

## Abstract

**Background:**

Food product labels based on the WHO 5-a-day fruit and vegetable (FV) message are becoming increasingly common, but these labels may impact negatively on complementary or subsequent FV consumption. This study aimed to investigate the impact of a ‘3 of your 5-a-day’ versus a ‘1 of your 5-a-day’ smoothie product label on subsequent FV consumption.

**Methods:**

Using an acute experimental design, 194 participants (90 males, 104 females) were randomised to consume a smoothie labelled as either ‘3 of your 5-a-day’ (*N* = 97) or ‘1 of your 5-a-day’ (*N* = 97) in full, following a usual breakfast. Subsequent FV consumption was measured for the rest of the day using 24-h recall. Usual FV consumption was also assessed via 24-h recall for the day before the study.

**Results:**

Regression analyses revealed a significantly lower subsequent FV consumption following smoothies displaying the ‘3 of your 5-a-day’ label compared to the ‘1 of your 5-a-day’ label (Beta = − 0.15, *p* = 0.04). Secondary analyses revealed these effects to be driven mainly by changes to consumption in usual high FV consumers, in females and in vegetable as opposed to fruit consumption.

**Conclusions:**

These findings demonstrate a role for label information in food intake, and the potential negative impacts of an exaggerated food product label on healthy food consumption and healthy dietary profiles.

## Background

There is a current trend for food manufacturers’ to add labels to food products to promote their contributions to health. One example of this is the addition of labels to fruit and vegetable (FV) food products based on contribution to World Health Organization and/or Government guidelines for health [1–3]. For example, in relation to the 5-a-day message in the UK [3], labels featuring ‘… of your 5-a-day’ are found on FV food products, and similar labels are found in other countries, based on local recommendations.

Evidence suggests that healthy food product labels can result in improved food product selection and purchasing [4–6]. More low-calorie and low-fat products, and less high-calorie and high-fat products were purchased in a canteen following the introduction of ‘high-’ and ‘low-’ ‘calorie’ and ‘fat’ labels for products [4], and more products labelled ‘healthy’ and less products labelled ‘unhealthy’ were purchased in a cafeteria setting over 3 months [5], and over one and two years [6].

This improved selection and purchasing can result in improved consumption at least to some degree [5, 6]. Limited evidence however, also suggests that healthy food product labels can result in reduced heathy food consumption. Participants consumed more cookies that were labelled ‘healthy’ than cookies that were labelled ‘gourmet’ in a laboratory taste test [7]. Consumers in an open buffet setting consumed more M&Ms labelled ‘low fat’ as opposed to ‘regular’ [8]. Consumers given a free ‘healthy’ food product in a fast food restaurant ordered more regular drinks and cookies, than consumers given a free ‘unhealthy’ food product [9]. Participants consumed more for lunch after a ‘low fat’ yoghurt preload than after a ‘high fat’ yoghurt preload in the laboratory [10], and participants consumed more for the total day after a ‘low fat’ lunch compared to a ‘high fat’ lunch [11]. These increased intakes are thought to result from a form of licencing, where adherence to a longer-term goal allows transgressions or indulgences that counter that longer-term goal [8, 9, 12–14]. Thus adhering to a low-fat diet through the consumption of low-fat products may allow greater consumption of those products compared to regular products, greater consumption of complementary non-low-fat products, or greater subsequent consumption in general.

Furthermore, where goals are specific and progress can be monitored, e.g. in the consumption of 5 portions of FV per day, early adherence to a goal can reduce later effort and subsequent later performance leading to a tendency to meet a goal rather than exceed it [15–17]. Thus, a food product label featuring a ‘2 of your 5-a-day’ message will likely result in the subsequent consumption of only up to three more FV portions that day as opposed to more than this, despite the scientific evidence reporting increasing health benefits with increasing FV consumption [18].

Thus, food product labels that highlight the healthy component of a product can have positive impacts of healthy dietary profiles, but licencing and goal adherence may reduce or negate these positive impacts. This situation is further worsened if the ‘healthy component’ portrayed in a food product label is exaggerated. The WHO 5-a-day FV guidelines generally recommend consideration of fruit juice as only one FV portion regardless of quantity, for reasons based on processing and fibre breakdown [1–3]. Published work from Australia, however, suggests exaggeration of FV portion contribution by some FV juice and FV drink product labels [19]. In the UK, the National Health Service currently recommends ‘*Unsweetened 100% fruit juice, vegetable juice and smoothies can only ever count as a maximum of one portion of your 5 A Day’* [3], and the British Dietetic Association states *‘A 150ml smoothie counts as one portion but some smoothies on the market today may contain two portions if they contain at least 150ml of fruit juice and at least 80g of crushed fruit or vegetable pulp’* [20], but many smoothies in the UK are labelled ‘2 of your 5-a-day’, without any inclusion of ‘crushed fruit’ in the ingredient list. Some recipes for home-made smoothies can also result in perceptions of 5-a-day contributions that are higher than the official one or two portions [21]. Exaggerated claims of the presence or proportion of FV provided by other food products were also found in the Australian study [19]. Few consumers furthermore may have the knowledge or inclination to look beyond a 5-a-day label claim. Few consumers report knowing or understanding the specifics of the various 5-a-day messages [21–23], and the different rules for single food products have been reported as particularly difficult [21, 23].

Whether through licencing or goal adherence, this study aimed to investigate the impact on subsequent FV consumption of a ‘3 of your 5-a-day’ versus a ‘1 of your 5-a-day’ FV product label. We hypothesised that the ‘3 of your 5-a-day’ label would result in a lower subsequent FV consumption compared to the ‘1 of your 5-a-day’ label.

## Methods

Using an independent groups design, participants received a smoothie labelled either ‘3 of your 5-a-day’ or ‘1 of your 5-a-day’ following a usual breakfast, and FV consumption for the rest of the day was measured.

### Participants

In total, 194 participants (90 male, 104 female, aged 18 years and over) took part. Participants were recruited from the staff and student population of Bournemouth University, UK and surrounding area, and the study ran between February and April 2016. All individuals who volunteered for the study were invited to take part, unless individuals stated known food allergies. Sample size was based on other recent studies using similar message- and label-based interventions [8, 9, 24], and the use of a wide sample aimed to increase the ecological validity of the study. The study was advertised as a study investigating ‘smoothie consumption in the morning’, with an interest in how this might affect intake for the rest of the day. No reference was made to the product labels.

### Food product labels

Two FV product labels were used. Each product label stated the product as a ‘smoothie’, included the smoothie flavour, and stated either ‘3 of your 5-a-day’ or ‘1 of your 5-a-day’, using the recognised national UK logo for the 5-a-day FV message. ‘3 of your 5-a-day’ or ‘1 of your 5-a-day’ labels were chosen as an exaggerated and a modest label respectively, and considering that most smoothies in the UK are marketed as ‘2 of your 5-a-day’ both labels would be perceived as equally incorrect. We did not include a ‘2 of your 5-a-day’ label to avoid confounding effects of the message with effects as a result of perceived accuracy / inaccuracy. Message labels were identical excepting the number of portions. No other information about the smoothies was provided on the smoothie labels. Ingredient and allergen information were provided and checked prior to inclusion in the study as part of the informed consent procedure. Labels were randomly allocated to participants by the participant drawing lots (equal chance of ether label), once each participant had provided consent and completed all initial measures. Three different smoothie flavours were used over the course of the study (*Tesco* ‘Blueberry’ Smoothie, *Tesco* ‘Mango and Passionfruit’ Smoothie, *Tesco* ‘Strawberry and Banana’ Smoothie, *Cheshunt, UK)*, all currently marketed as ‘2 of your 5-a-day’. Each smoothie was provided in a refrigerated 250 ml bottle, and all participants had a choice of one of two flavours.

### Subsequent FV consumption

Subsequent FV consumption was assessed using 24-h recalls. The day after smoothie consumption, participants were asked to recall all foods consumed the previous day, using household portion sizes. The 24-h recall method was used as an accurate measure of food intake over a single day, and a measure of food intake reported as reliable for measuring FV intakes [25, 26]. Food recalls were conducted by the researcher and checked at the time of completion for completeness and accuracy, using prompts for frequently missed food items, such as snacks, condiments, and drinks with meals [25, 26]. Food recalls were subsequently coded by the researcher for portions of fruit and portions of vegetables consumed based on current UK portion size guidelines [3]. All researchers who administered food recalls were trained in food recall methodology prior to the study and were familiar with current UK FV portions size guidelines [3].

Subsequent FV consumption was highly likely to be influenced by usual FV consumption [24, 27–29]. Usual FV consumption was assessed again using a 24-h recall completed for the day before smoothie consumption. This recall was completed on study entry, prior to smoothie consumption, and also served to familiarise participants with the 24-h recall procedure.

### Procedure

The study was conducted in the Eating Behaviours Laboratory at Bournemouth University, Bournemouth, UK, in February–April, 2016. Participants were asked to attend the Laboratory between 8 am–11 am, following their usual breakfast, on two consecutive study days. On the first study day, participants first completed the 24-h recall as a measure of usual consumption, and secondly, were provided with and asked to consume a labelled smoothie of their choice. All participants were asked to look at their smoothie before consumption and were required to consume their smoothie in full, although neither the label nor the message were mentioned. Attention was not specifically drawn to the label or the 5-a-day message to more closely represent a real-world consumption scenario and increase the ecological validity of the study. Participants were also unaware of the alternate condition and label in the study. While participants, thus, were not blinded to study condition, biases in reporting were unlikely to be systematic between conditions. On the following day, participants were asked to return to the Laboratory at the same time, again completed the 24-h food recall, and were debriefed. The study took 20–30 min on each day to complete.

### Analyses

All participants completed the study, and there were no missing data. Analyses were conducted on an Intention-to-Treat basis. Data were analysed by multiple linear regression, where subsequent FV consumption was firstly predicted by label type (model 1), and secondly also by usual FV consumption and gender (model 2). The second analyses were conducted to investigate the relative impact of the label compared to usual consumption. Gender was included following reports that both usual FV consumption and the impacts of food labels are typically higher in females compared to males [30–33]. Analyses were also conducted to explore whether usual high or low consumers and whether males or females were differentially affected by the labels [30–33]. These analyses investigated the impact of interactions between usual FV consumption and label type (FV consumption * label) (model 3) and gender and label type (gender * label) (model 4). In these regression models, subsequent FV consumption was predicted using the interaction term and the remaining predictor (gender and usual FV consumption respectively). Significant interaction terms would demonstrate that usual high or low consumers and/or that males or females were differentially affected by the labels taking other variables into account. Analyses were conducted for FV consumption, and for fruit consumption and vegetable consumption separately [34].

## Results

In total, 97 participants received a smoothie with the ‘3 of your 5-a-day’ label (46 males, 51 females), and 97 participants received a smoothie with the ‘1 of your 5-a-day’ label (44 males, 53 females). No other demographic details for participants were collected.

### FV consumption

Mean (sd.) subsequent FV consumption for the ‘3 of your 5-a-day’ group was 1.8 (1.6) FV portions, and for the ‘1 of your 5-a-day’ group was 2.3 (2.0) FV portions. A split of the numbers of participants consuming 0, 0.5–1, 1.5–2, 2.5–3, 3.5–4, 4.5–5 and 5.5 plus FV portions subsequently in each group is given in Table 1. Daily FV consumption was significantly lower in consumers who received the ‘3 of your 5-a-day’ label compared to those who received the ‘1 of your 5-a-day’ label (*R* = 0.15, R^2^ = 0.02, adj. R^2^ = 0.02, F(1,193) = 4.42, *p* = 0.04; Beta = − 0.15, *p* = 0.04).

**Table 1:** Number of participants consuming 0–5.5 + subsequent FV portions in response to the ‘1 of you 5-a-day’ label and the ‘3 of your 5-a-day’ label (*N* = 194)

Subsequent FV portions	0	0.5–1	1.5–2	2.5–3	3.5–4	4.5–5	5.5 +
1 of your 5-a-day (*N* = 97)	15	25	21	11	11	8	6
3 of your 5-a-day (*N* = 97)	21	29	20	12	10	3	2

On consideration also of usual consumption and gender, the regression model remained significant (*R* = 0.63, R^2^ = 0.40, adj. R^2^ = 0.39, F(3,193) = 42.03, *p* < 0.01), but label type was no longer a significant predictor (Beta = − 0.01, *p* = 0.09). Higher subsequent FV consumption was instead associated with higher usual FV consumption (Beta = 0.56, *p* < 0.01), and being female (Beta = − 0.18, *p* < 0.01).

Consideration of the interaction terms revealed greater effects of the labels in those of higher usual FV consumption (Beta = 0.34, *p* < 0.01), and in females (Beta = − 0.17, *p* < 0.01). Mean (sd.) subsequent and usual FV consumption in high and low FV consumers and in male and female consumers in response to the ‘3 of your 5-a-day’ label and the ‘1 of your 5-a-day’ label are given in Tables 2 and 3 respectively.

**Table 2:** Mean (sd.) (unadjusted) subsequent and usual FV consumption in high and low FV consumers in response to the ‘1 of you 5-a-day’ label and the ‘3 of your 5-a-day’ label (*N* = 194)

	Label type	Usual FV consumption	Subsequent FV consumption
Low usual FV consumers	1 of your 5-a-day (*N* = 60)	1.0 (0.8)	1.5 (1.5)
	3 of your 5-a-day (*N* = 63)	0.9 (0.8)	1.3 (1.5)
High usual FV consumers	1 of your 5-a-day (*N* = 37)	4.4 (1.5)	3.7 (1.9)
	3 of your 5-a-day (*N* = 34)	4.0 (1.1)	2.6 (1.5)

**Table 3:** Mean (sd.) (unadjusted) subsequent and usual FV consumption in male and female consumers in response to the ‘1 of you 5-a-day’ label and the ‘3 of your 5-a-day’ label (*N* = 194)

	Label type	Usual FV consumption	Subsequent FV consumption
Females	1 of your 5-a-day (*N* = 53)	2.5 (2.1)	2.7 (2.3)
	3 of your 5-a-day (*N* = 51)	2.4 (1.9)	2.3 (1.8)
Males	1 of your 5-a-day (*N* = 44)	2.0 (1.9)	1.8 (1.4)
	3 of your 5-a-day (*N* = 46)	1.5 (1.5)	1.2 (1.1)

### Fruit consumption

No effects of label type were found on fruit consumption (Primary model: *R* = 0.10, R^2^ = 0.01, adj. R^2^ = 0.01, F(1,193) = 1.78, *p* = 0.18; Beta = − 0.10, *p* = 0.18; Secondary model: *R* = 0.52, R^2^ = 0.27, adj. R^2^ = 0.26, F(3,193) = 23.68, *p* < 0.01; Beta = − 0.06, *p* = 0.38). Effects of usual fruit consumption and gender were found, as above (smallest Beta = − 0.19, *p* < 0.01). Interactions between label type and usual fruit consumption and gender were also found, as above (smallest Beta = − 0.14, *p* = 0.03).

### Vegetable consumption

Vegetable consumption was significantly lower following the ‘3 of your 5-a-day’ label compared to the ‘1 of your 5-a-day’ label (*R* = 0.19, R^2^ = 0.04, adj. R^2^ = 0.03, F(1,193) = 6.95, *p* = 0.01; Beta = − 0.19, *p* = 0.01). On consideration also of usual consumption and gender, the model remained significant (*R* = 0.61, R^2^ = 0.38, adj. R^2^ = 0.37, F(3,193) = 38.36, *p* < 0.01), but the effects of label type were again reduced (Beta = − 0.16, *p* = 0.02), and higher subsequent vegetable consumption was also associated with higher usual vegetable consumption (Beta = 0.53, *p* < 0.01), and being female (Beta = − 0.16, *p* = 0.01). On consideration of the interaction terms, greater effects of the labels were again found in those of higher usual vegetable consumption (Beta = 0.34, *p* < 0.01), and in females (Beta = − 0.21, *p* < 0.01).

Results of all regression analyses are provided in Table 4.

**Table 4:** Beta co-efficients and their significance in all regression analyses, predicting subsequent FV consumption, fruit consumption and vegetable consumption (*N* = 194)

Model 1		Model 2		Model 3		Model 4	
Predictors	Beta value, significance	Predictors	Beta value, significance	Predictor	Beta value, significance	Predictors	Beta value, significance
Subsequent FV consumption							
Label^a^	β = − 0.15, *p* = 0.04	Label^a^	β = − 0.10, *p* = 0.09	Label^a^ x usual FV consumption	β = 0.34, *p* < 0.01	Label^a^ x Gender^b^	β = − 0.17, *p* < 0.01
		Usual FV consumption	β = 0.56, *p* < 0.01	Gender^b^	β = − 0.23, *p* < 0.01	Usual FV consumption	β = 0.57, *p* < 0.01
		Gender^b^	β = − 0.18, *p* < 0.01				
Subsequent fruit consumption							
Label^a^	β = − 0.10, *p* = 0.18	Label^a^	β = − 0.06, *p* = 0.38	Label^a^ x usual FV consumption	β = 0.28, *p* < 0.01	Label^a^ x Gender^b^	β = − 0.14, *p* = 0.03
		Usual FV consumption	β = 0.45, *p* < 0.01	Gender^b^	β = − 0.22, *p* < 0.01	Usual FV consumption	β = 0.46, *p* < 0.01
		Gender^b^	β = − 0.19, *p* < 0.01				
Subsequent vegetable consumption							
Label^a^	β = − 0.19, *p* = 0.01	Label^a^	β = − 0.14, *p* = 0.02	Label^a^ x usual FV consumption	β = 0.34, *p* < 0.01	Label^a^ x Gender^b^	β = − 0.21, *p* < 0.01
		Usual FV consumption	β = 0.53, *p* < 0.01	Gender^b^	β = − 0.21, *p* < 0.01	Usual FV consumption	β = 0.54, *p* < 0.01
		Gender^b^	β = − 0.16, *p* = 0.01				

^a^Label variable – ‘1 of your 5-a-day’ label vs ‘3 of your 5-a-day’ label, coded 1 and 3 respectively

^b^Gender variable – female vs male, coded 1 and 2 respectively

## Discussion

Several key findings emerge from this study. Firstly, exposure to a ‘3 of your 5-a-day’ FV label following a usual breakfast resulted in reduced subsequent FV consumption compared to exposure to a ‘1 of your 5-a-day’ label. These findings mirror early findings where healthy food labels were found to result in reduced subsequent healthy food consumption [10, 11]. These effects are most plausibly explained either as a licencing effect [8, 9, 12–14], where consumers allow themselves lower adherence to a dietary goal (consuming 5 FV a day) for the rest of the day as a result of their perceived earlier adherence to that goal, or as a goal adherence effect [15–17], where consumers consume healthy foods to a healthy food goal and no further, or as both. We cannot disentangle these two mechanisms in this study. We suggest however, that our effects are predominantly due to licencing. Effects due to goal adherence would have resulted in consumers in the ‘3 of your 5-a-day’ group consuming 2 FV portions following the smoothie. However, 19 consumers in the ‘3 of your 5-a-day’ group consumed 2 FV portions following the smoothie, and this was no higher than the number that would have occurred by chance assuming equal distribution over 0–5 FV portions (*n* = 16), than the number of consumers that usually consumed 2 FV portions / day based on intake measures of the previous day (*n* = 15), or than the number of consumers consuming 2 FV portions in the ‘1 of your 5-a-day’ group (*n* = 20) (largest χ^2^ = 1.1, df = 1, *p* > 0.05). An element of goal adherence may still have occurred to some degree, or in some individuals, and further investigation of mechanisms would clearly be of interest.

In a practical sense, these findings demonstrate a danger of providing information suggesting exaggerated FV provision on food product labels. FV consumption is known to be lower than WHO recommendations in Europe and the US [35, 36], and the levels of usual FV consumption reported in this study (mean (sd.) = 2.1 (1.9) portions of FV / day) are comparable with these reports [35, 36]. The provision of a free smoothie resulted in an increase in mean consumption to 3.0 (1.8) FV portions on the following day, but individuals receiving a smoothie with the ‘3 of your 5-a-day’ label would have perceived their consumption at 5.0 (1.8) FV portions. Based on suggestions of goal-adherence, these consumers are unlikely to consume further FV on this day. Based on licencing, consumers may easily excuse themselves from consuming more. The detrimental impacts on FV consumption of the exaggerated message would suggest greater regulation may be required to ensure accuracy in these types of messages. Regulation is currently in place across Europe, the US and Australia on necessary food product information, such as the presence of allergens, and food product claims (e.g. [37–40]), but the specifics of these regulations can result in labels that misinform consumers [19]. We have no reason furthermore, to consider our findings to be specific to FV food product labels or UK consumers. Effects as a result of exaggerated claims may be more obvious for 5-a-day FV message labels, due to the use of a clear goal, and a goal that is well-known, but the same principles may be equally applicable to any dietary goal.

Secondly, these effects were reduced by consideration also of usual FV consumption and gender. These findings suggest firstly, that usual FV consumption and gender are more important predictors of subsequent FV consumption than the label provided, and secondly that the effects due to label are, at least partially, a result of some interaction between label, usual FV consumption and gender. The higher FV consumption in the study by usual high consumers and by females is unsurprising. Repeated previous research demonstrates the importance of habit or usual practices in FV consumption [24, 27–29], and the usual higher consumption of FV by females compared to males [30, 33].

The interaction effects reveal differential effects of the label dependent on usual FV consumption and gender. These findings are noteworthy. Different effects of food labels on different consumers has previously been suggested elsewhere [7, 9, 14, 41, 42]. In our study, greater effects of the label were found in usual high consumers of FV and in females. These findings may suggest that high consumers of FV and females consume high amounts of FV as a result of deliberate portion counting and other cognitive methods. Various work suggests a high cognitive input into healthy eating including FV consumption [28, 29, 43–45], and that females are more inclined to use these cognitive methods than males [46, 47]. Work on the cognitive controls of eating behaviour, and particularly work on dietary restraint, suggests that these cognitive methods can be effective, but can also be prone to disruption through environmental or emotional cues [10, 11, 24, 46–49]. The findings of this study could demonstrate a clear disruption to cognitively controlled eating behaviour as a result of (mis) information.

Effects in FV consumption were also largely driven by effects in vegetable consumption not in fruit consumption. These effects are surprising given that the smoothies used in the study were predominantly fruit smoothies, but various evidence suggests that individuals tend to find it easier to consume fruit than vegetables [34, 50]. Thus, while the 5-a-day message relates to fruit and vegetables together, any additional FV consumption may have an impact on the more difficult (vegetable consumption) as opposed to the easier (fruit consumption) goal. These findings may also demonstrate a disadvantage of the combination of fruit and vegetables in the WHO 5-a-day message. Recognition of the health benefits and the low consumption of vegetables specifically has resulted in the use of separate messages for fruits and for vegetables by certain Governments [34].

Comparable to our effects on fruit consumption, other studies also demonstrate no effects on intake as a result of food product labels [41, 42, 51]. These studies suggest greater impacts on intake as a result of other properties of a food product, such as texture [51], or suggest an important role for individual differences [41, 42], including socio-demographic [7, 42] or personality characteristics [9, 14, 42]. Our study is limited by our lack of assessment of these other characteristics of potential influence, although randomisation procedures should have ensured that any bias was unsystematic. We also took no assessment of participant’s perceptions of the smoothies. It is possible that the smoothie with a ‘3 of your 5-a-day’ label was perceived to be larger or more energy dense than the alternate smoothie, and so may have resulted in a lower intake of all foods. However, given the assessment of food intake over a whole day, and a usual very limited compensation for small amounts of energy over a whole day [49], we consider this possibility unlikely.

Our study is also limited by the use of predominantly educated individuals, and it can be argued that food product labels may be of greater value in less educated individuals [52]. We also did not use a ‘no label’ control to investigate the effects of labels per se. Investigation of the effects of a label vs no label was not the purpose of this study. Other studies suggest that labels can improve dietary profiles compared to no labels [52, 53], although small and limited impacts in general have also been suggested [54].

Finally, as an unintended outcome, our study also demonstrates an impact of providing a free fruit smoothie on daily FV consumption. Mean total daily FV consumption increased from a mean (sd.) of 2.1 (1.9) portions of FV / day) on the day before consuming the smoothie (usual consumption) to a mean (sd.) daily consumption of 3.0 (1.8) FV portions when a smoothie was provided. Other studies demonstrate increased consumption as a result simply of increased provision [55–58], and this study confirms increased provision as a strategy for increasing FV consumption.

## Conclusions

In conclusion, consumption of a smoothie sporting a ‘3 of your 5-a-day’ food product label compared to a ‘1 of your 5-a-day’ food product label resulted in reduced subsequent FV consumption, and this effect was largely driven by changes in consumption in usual high FV consumers, in females, and in vegetable as opposed to fruit consumption. Effects are likely driven by cognitive controls of food intake, such that perceived earlier adherence to a healthy dietary goal results in licenced transgressions or reduced efforts towards reaching that goal. The findings demonstrate a potential negative role for healthy food product labels in healthy dietary consumption.
